# Non‐alcoholic fatty liver disease prevalence in Australia has risen over 15 years in conjunction with increased prevalence of obesity and reduction in healthy lifestyle

**DOI:** 10.1111/jgh.16314

**Published:** 2023-08-12

**Authors:** Karl Vaz, William Kemp, Ammar Majeed, John Lubel, Dianna J Magliano, Kristen M Glenister, Lisa Bourke, David Simmons, Stuart K Roberts

**Affiliations:** ^1^ Department of Gastroenterology and Hepatology Alfred Health Melbourne Victoria Australia; ^2^ Central Clinical School Monash University Melbourne Victoria Australia; ^3^ Diabetes and Population Health Baker Heart and Diabetes Institute Melbourne Victoria Australia; ^4^ Department of Rural Health University of Melbourne Melbourne Victoria Australia; ^5^ School of Medicine, Macarthur Clinical School Western Sydney University Melbourne Victoria Australia

**Keywords:** epidemiology, NAFLD, prevalence

## Abstract

**Background and Aim:**

Non‐alcoholic fatty liver disease (NAFLD) is the most prevalent liver condition globally. The aim of this study was to evaluate the change in age‐ and sex‐standardized prevalence of NAFLD in regional Victoria over a 15‐year period and explore the underlying factors associated with differences over time.

**Methods:**

Repeated comparative cross‐sectional studies in four towns in regional Victoria, Australia. Individuals randomly selected from households from residential address lists from local government organizations in 2001–2003 (CrossRoads I [CR1]) and 2016–2018 (CrossRoads II [CR2]) with 1040 (99%) and 704 (94%) participants from CR1 and CR2 having complete data for analysis. Primary outcome was change in prevalence estimates of NAFLD (defined by a fatty liver index ≥ 60 in the absence of excess alcohol and viral hepatitis) between 2003 and 2018.

**Results:**

Crude prevalence of NAFLD increased from 32.7% to 38.8% (*P* < 0.01), while age‐standardized/sex‐standardized prevalence increased from 32.4% to 35.4% (*P* < 0.01). Concurrently, prevalence of obesity defined by BMI and elevated waist circumference increased 28% and 25%, respectively. Women had a greater increase in the prevalence of NAFLD than men, in parallel with increasing prevalence of obesity. Proportion of participants consuming takeaway food greater than once weekly increased significantly over time. Up to 60% of NAFLD patients require additional tests for assessment of significant fibrosis.

**Conclusions:**

Crude and age‐standardized/sex‐standardized prevalence of NAFLD have both increased significantly over the last 15 years, particularly among women, in association with a parallel rise in the prevalence of obesity.

## Introduction

Non‐alcoholic fatty liver disease (NAFLD) is the most prevalent condition impacting the liver with an estimated 25–30% of adults affected globally^1^, ^2^ and is rapidly emerging as the foremost indication for liver transplantation.^3^, ^4^ The widely accepted driver for rising prevalence is the increase in the prevalence of obesity and concomitant insulin resistance.^5^, ^6^

Among Organization for Economic Co‐operation and Development countries, Australia ranked within the top 10 for obesity prevalence in 2016.^7^ The prevalence of NAFLD in Australia has been forecast to increase by 25% over a decade using a Markov based model.^8^ However, the model was reliant on imputed prevalence extrapolated from studies conducted outside Australia, leading to uncertainty in the results.

Despite the obesity epidemic gripping Australia, there is a dearth of NAFLD epidemiologic data from Australia. Prevalence studies are important to forecast future events, allowing public health policy to be better informed when allocating limited resources in the most efficient manner.

The aim of this study is to evaluate the change in age‐ and sex‐standardized prevalence of NAFLD over a 15‐year period and explore the underlying factors associated with differences over time. We hypothesize that the age‐ and sex‐standardized NAFLD prevalence has increased in association with a rise in obesity prevalence.

## Methods

This study is an analysis from the CrossRoads I and II studies (CR‐1 [2001–2003] and CR‐2 [2016–2018], respectively) conducted across the four towns in both studies (Shepparton‐Mooroopna [regional center], and Benalla, Cobram, and Seymour [rural towns]) in the Goulburn Valley, Victoria. Methodology from the original studies has previously been published in detail.^9^ To summarize, the CrossRoads studies were repeated cross‐sectional, population‐based studies that randomly selected households to undertake a health questionnaire of each household's residents, with one adult (≥ 18 years old) from each household invited to participate in a clinic sub‐study. The regional center *versus* rural town recruitment was 2:1 in CR‐1 and 1:1 in CR‐2. The clinic sub‐study collected detailed information on demographics, clinical information via a series of health‐related questionnaires, anthropometry, vital signs, and laboratory measures from participants. Participants with insufficient information to determine NAFLD were excluded from this analysis.

### Definitions

Fatty liver was diagnosed according to fatty liver index (FLI) score ≥ 60, calculated from body mass index (BMI), waist circumference, gamma‐glutamyl transferase (GGT), and fasting triglycerides.^10^ NAFLD was considered in those with fatty liver in the absence of significant alcohol consumption (≥ 210 g/week in men, ≥ 140 g/week in women^11^; determined through a detailed questionnaire) or viral hepatitis (self‐report in CR‐1; serology for chronic hepatitis B and C in CR‐2).

Metabolic syndrome was defined according to criteria jointly agreed upon by a group of international societies.^12^ Elevated waist circumference considered individual thresholds for those of Asian descent (≥ 90 cm in men, ≥ 80 cm in women) and all other ethnicities (≥ 102 cm in men, ≥ 88 cm in women). Overweight and obesity were defined as BMI ≥ 25 kg/m^2^ and ≥ 30 kg/m^2^, respectively; hypertension as systolic blood pressure ≥ 130 mmHg and/or diastolic blood pressure ≥ 85 mmHg or on anti‐hypertensive therapy; and dyslipidaemia according to lipid parameters as per the Australian Institute of Health and Welfare.^13^

Sufficient physical activity was considered as ≥ 150 min/week of dedicated exercise, as recommended by the Australian Government Department of Health and Aged Care.^14^ Diet adequacy was determined according to consuming at least four serves of vegetables daily and two serves of fruit daily as per Australian dietary guidelines,^15^ with a more inclusive threshold of four serves of vegetables as this was coded together with five serves on diet questionnaire. Healthy lifestyle was defined as those subjects who participated in sufficient physical activity together with having an adequate diet.

Commonly used non‐invasive tests (NITs) for liver fibrosis—Fibrosis‐4 index (FIB‐4) and NAFLD Fibrosis Score (NFS)—were calculated to stage fibrosis, including their adopted cut‐offs for low‐risk, indeterminate‐risk, and high‐risk for advanced fibrosis/cirrhosis.^16^, ^17^

### Statistical analysis

Categorical data are presented as frequency and percentages with between group comparisons conducted with *χ* ^2^ test or Fisher's exact test. Continuous data are presented as mean (standard deviation) or median (interquartile range) and comparisons made through Student's *t*‐test or Mann–Whitney *U* test for parametric and non‐parametric data, respectively, following normality assessment of each covariate. Age‐standardized and gender‐standardized prevalence was calculated using direct standardization, with Shepparton 2016 census data as the reference population.^18^ Two‐tailed *P*‐value <0.05 is considered statistically significant. All statistical analysis was conducted using IBM Statistical Package for the Social Sciences (SPSS) Statistics, version 28.0.0.0 and figures produced through Prism GraphPad Version 9.4.1.

## Results

In total, 1040 (99.2%) and 704 (94.2%) participants from CR‐1 and CR‐2 clinic sub‐studies, respectively, had sufficient evaluable data for analysis. Participants included and excluded in the analysis were comparable across demographic, clinical, laboratory and lifestyle parameters (Table S1). Compared with CR‐1, participants in CR‐2 were older, from a more ethnically diverse background, and with a higher proportion living in rural towns, having completed secondary school or further study, and with private health insurance (Table 1). There were slightly more women than men in each study, but the gender distribution remained the same.

**Table 1:** Difference in baseline demographics in entire cohort between CrossRoads studies

Variable	CrossRoads 1 (*n* = 1040)	CrossRoads 2 (*n* = 704)	*P*‐value
***Demographic***			
Male	460 (44.2%)	314 (44.6%)	0.88
Age, years	52.8 (15.7)	59.1 (16.1)	**<0.01**
Australian‐born	910 (87.9%)	596 (84.8%)	0.06
Ethnic background			**<0.01**
White	1011 (97.4%)	654 (92.9%)	
Asian	11 (1.1%)	30 (4.3%)	
Aboriginal and Torres Straits Islander	7 (0.7%)	6 (0.9%)	
Other	9 (0.9%)	14 (2.0%)	
Regional town			**<0.01**
Shepparton/Mooroopna	690 (66.3%)	331 (47.0%)	
Benalla	120 (11.5%)	150 (21.3%)	
Cobram	116 (11.2%)	109 (15.5%)	
Seymour	114 (11.0%)	114 (16.2%)	
Private health insurance	498 (47.9%)	434 (61.6%)	**<0.01**
Education secondary school and beyond	488 (47.1%)	439 (62.4%)	**<0.01**
***Clinical***			
Weight, kg	78.8 (16.7)	82.2 (19.2)	**<0.01**
BMI, kg/m^2^	27.9 (5.4)	29.7 (19.6)	**0.02**
BMI, kg/m^2^			**0.01**
< 25	331 (31.8%)	182 (25.9%)	
25 to < 30	420 (40.4%)	272 (38.6%)	
≥ 30	289 (27.8%)	250 (35.5%)	
Waist circumference, cm	94.7 (14.4)	98.5 (15.1)	**<0.01**
Elevated waist circumference	501 (48.2%)	423 (60.1%)	**<0.01**
Hypertension	578 (55.6%)	384 (54.9%)	0.77
Dyslipidemia	653 (63.8%)	402 (57.8%)	**0.01**
Type 2 diabetes mellitus	76 (7.3%)	64 (9.4%)	0.12
Metabolic syndrome	342 (32.9%)	252 (36.3%)	0.15
***Lifestyle factors***			
Alcohol excess	166 (16.0%)	99 (14.1%)	0.28
Smoking status			**<0.01**
Current smoker	179 (17.3%)	65 (9.5%)	
Ex‐smoker	345 (33.3%)	233 (34.1%)	
Non‐smoker	513 (49.5%)	385 (56.4%)	
Physical activity, minutes/week	278 (293)	265 (229)	0.40
Adequate physical activity	480 (66.9%)	329 (66.1%)	0.78
Adequate diet	228 (22.0%)	133 (19.5%)	0.21
Takeaway food ≥ once per week	271 (26.1%)	210 (30.7%)	**0.04**
Healthy lifestyle	135 (18.8%)	85 (17.1%)	0.43
***Laboratory measured risk factors***			
GGT, U/L	34 (41)	33 (39)	0.48
ALT, U/L	25 (19)	26 (16)	0.31
ALT ≥ 1.5× upper limit of normal*	168 (16.2%)	107 (15.2%)	0.59
AST, U/L	26 (10)	26 (9)	0.63
Fasting glucose, mmol/L	5.4 (1.3)	5.3 (1.2)	0.31
HbA1c, %	5.3 (0.6)	5.5 (0.7)	**<0.01**
Total cholesterol, mmol/L	5.3 (1.0)	4.9 (1.0)	**<0.01**
LDL, mmol/L	3.2 (0.9)	2.8 (0.9)	**<0.01**
HDL, mmol/L	1.4 (0.4)	1.4 (0.4)	0.72
Low HDL**	288 (27.7%)	215 (30.5%)	0.20
Elevated triglycerides***	342 (32.9%)	220 (31.3%)	0.47
FIB‐4	1.25 (0.77)	1.31 (0.69)	0.11
FIB‐4 categorical			**<0.01**
< 1.30	676 (65.0%)	372 (57.8%)	
1.30 to 2.67	316 (30.4%)	249 (38.7%)	
> 2.67	48 (4.6%)	23 (3.6%)	
NAFLD Fibrosis Score (NFS)	−1.648 (1.406)	−1.415 (2.350)	**0.01**
NFS categorical			**<0.01**
<−1.455	606 (58.3%)	313 (50.4%)	
−1.455 to 0.676	379 (36.5%)	276 (44.4%)	
>0.676	55 (5.3%)	32 (5.2%)	
***Outcome***			
NAFLD	340 (32.7%)	273 (38.8%)	**<0.01**

All continuous parameters presented as mean (SD); all categorical parameters presented as *n* (%).

BMI, body mass index; GGT, gamma‐glutamyl transferase; ALT, alanine aminotransferase; AST, aspartate aminotransferase; HbA1c, glycosylated hemoglobin; LDL, low density lipoprotein; HDL, high density lipoprotein; FIB‐4, fibrosis‐4 index; NAFLD, non‐alcoholic fatty liver disease.

† Upper limit normal = 30 U/L men and 20 U/L women.

‡ Low HDL = < 1.0 mmol/L in men or < 1.3 mmol/L in women or on lipid‐lowering therapy.

§ Elevated triglycerides = ≥ 1.70 mmol/L.

### Prevalence

**Figure 1:**
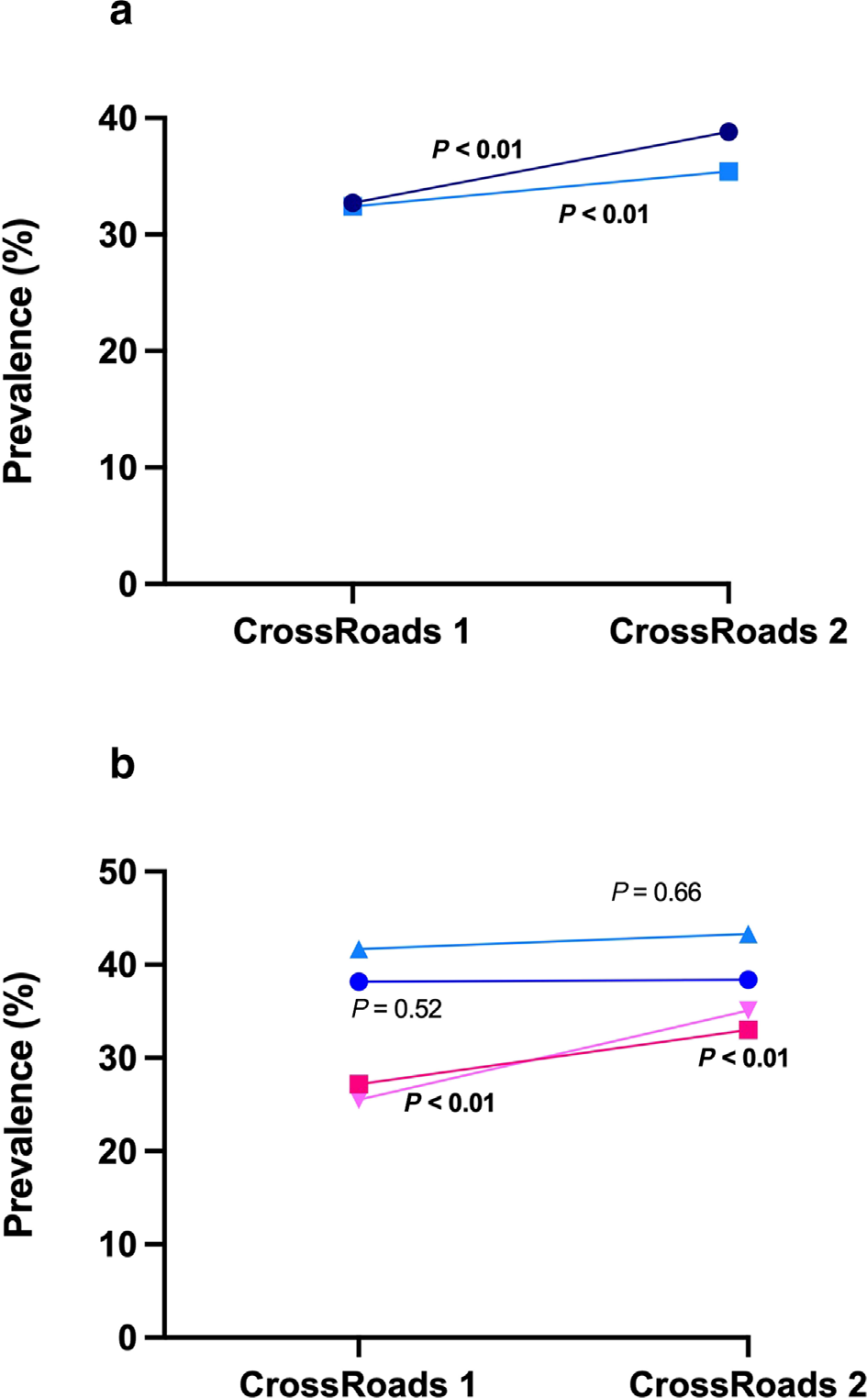
Changes in NAFLD prevalence. (A) Entire cohort. 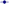, NAFLD crude; 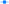, NAFLD standardized. (B) according to gender. 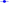, NAFLD male standardized; 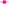, NAFLD female standardized; 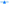, NAFLD male crude; 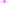, NAFLD female crude.

**Figure 2:**
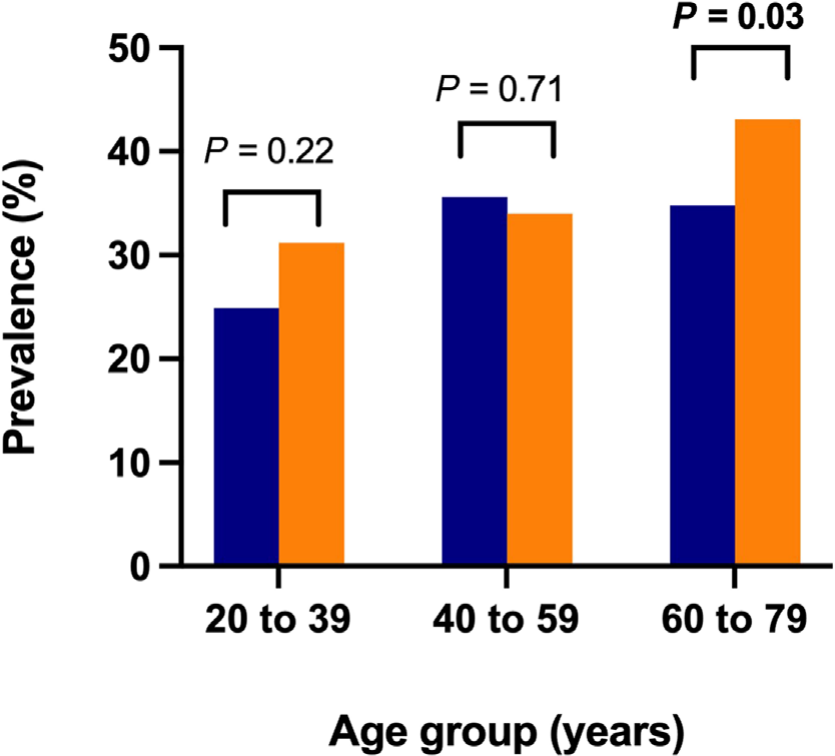
Changes in NAFLD prevalence among different age groups. 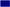, CrossRoads 1; 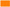, CrossRoads 2.

Crude NAFLD prevalence increased significantly between CR‐1 and CR‐2, from 32.7% to 38.8% (*P* < 0.01). There was a significant, albeit attenuated increase in age−/sex‐standardized prevalence, from 32.4% (95% confidence interval [CI] 29.5–35.4) to 35.4% (95% CI 31.3–39.5) (*P* < 0.01) (Table 1, Fig. 1a). NAFLD prevalence was higher in men than women across both studies but whereas the crude and age‐standardized prevalence remained stable in men over time (crude: 41.7% to 43.3%, *P* = 0.66; age‐standardized: 38.2% [95% CI 33.9–42.4] to 38.4% [95% CI 31.9–44.8], *P* = 0.52), it significantly increased in women (crude: 25.5% to 35.1%, *P* < 0.01; age‐standardized: 27.2% [95% CI 23.1–31.4] to 33.0% [95% CI 28.0–38.1], *P* < 0.01) (Fig. 1b). The greatest increase in NAFLD prevalence was in those aged between 60 to 79 years old (34.8% to 43.1%, *P* = 0.03) (Fig. 2). There was no difference in crude NAFLD prevalence between regional and rural centers, including similar rates of rise between studies, however upon standardization, there was a significantly higher prevalence in rural towns, albeit with wide and overlapping confidence intervals (Table S2).

On subgroup analysis, the crude NAFLD prevalence was stable over time in those with overweight/obesity and type 2 diabetes mellitus (T2DM, 46.8% to 51.5%, *P* = 0.10 and 60.5% to 64.1%, *P* = 0.67, respectively). Assessing both cohorts together, those with overweight/obesity and T2DM had a significantly higher prevalence of NAFLD than those without these risk factors (48.8% *vs* 2.3%, *P* < 0.01 and 62.1% *vs* 32.7%, *P* < 0.01, respectively).

### Factors associated with increasing non‐alcoholic fatty liver disease prevalence

**Figure 3:**
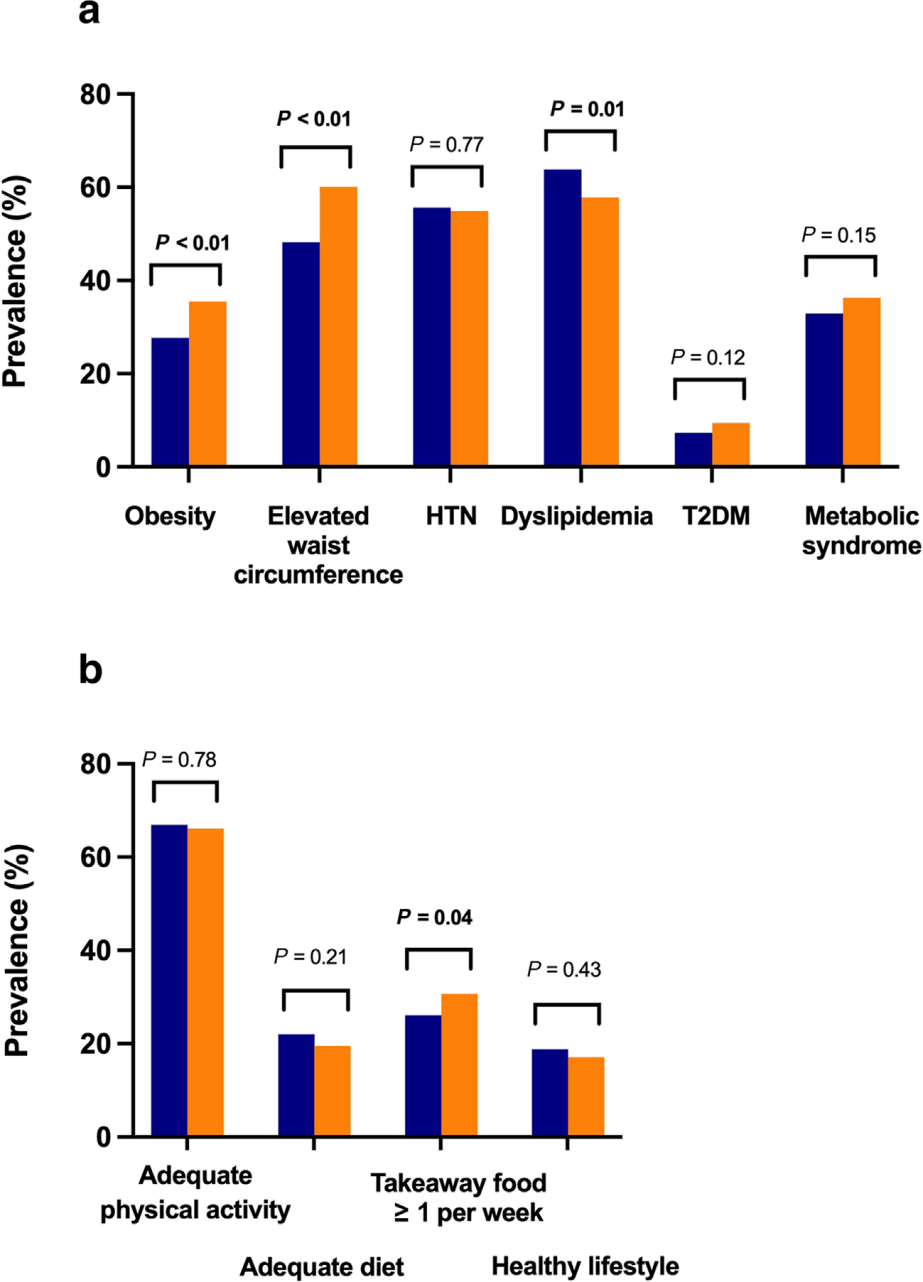
Changes in (A) crude prevalence of metabolic risk factors. Changes in (B) crude prevalence of lifestyle factors. 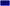, CrossRoads 1; 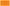, CrossRoads 2.

Mean BMI and waist circumference significantly increased over time, with a 28% and 25% relative rise in obesity and elevated waist circumference, respectively (Table 1, Fig. 3a). There was an increase in crude prevalence of T2DM and metabolic syndrome, while prevalence of hypertension was stable, and dyslipidemia was the only risk factor to decrease. This largely held true for age‐standardized/sex‐standardized values of metabolic risk factors, particularly for rise in obesity and elevated waist circumference (Table 2). Among dietary and lifestyle factors, the proportion of subjects consuming takeaway food at least once per week rose from 26.1% to 30.7% (*P* = 0.04), while there was a non‐significant decrease in the number consuming an adequate diet and leading a healthy lifestyle (*P* = 0.21 and *P* = 0.43, respectively) (Table 1, Fig. 3b). There was no change in the prevalence of individuals participating in adequate physical activity. These differences were accentuated upon standardization, particularly for increase in takeaway food consumption and reduction in healthy lifestyle (Table 2).

**Table 2:** Age‐standardized/sex‐standardized prevalence of metabolic and lifestyle risk factors

Variable	CrossRoads 1	CrossRoads 2	*P*‐value
Obesity	29.9 (27.1–32.7)	33.5 (29.4–37.6)	**<0.01**
Elevated waist circumference	50.3 (47.3–53.4)	54.8 (50.9–58.7)	**<0.01**
Hypertension	55.6 (53.0–58.2)	50.7 (46.5–54.8)	**<0.01**
Dyslipidemia	61.7 (58.7–64.7)	53.8 (49.6–58.0)	**<0.01**
Type 2 diabetes mellitus	7.6 (5.9–9.2)	7.7 (5.7–9.8)	0.22
Metabolic syndrome	33.5 (30.6–36.3)	32.2 (28.6–35.8)	**<0.01**
Adequate physical activity	65.4 (61.5–69.2)	62.7 (57.5–67.9)	**<0.01**
Adequate diet	20.9 (18.4–23.4)	16.5 (13.3–19.6)	**<0.01**
Takeaway food ≥ once per week	24.6 (22.1–27.2)	39.1 (34.9–43.3)	**<0.01**
Healthy lifestyle	17.3 (14.6–20.0)	13.9 (10.7–17.2)	**<0.01**
Data presented as percentage (95% confidence interval)			

### Gender differences in changes to risk factors

Crude prevalence of obesity (28.5% *vs* 39.2%, *P* < 0.01) and elevated waist circumference (49.8% *vs* 67.2%, *P* < 0.01) significantly increased in women over time, while there was no change in prevalence in either risk factor in men (obesity 27.0% *vs* 30.9%, *P* = 0.23; elevated waist circumference 46.1% *vs* 51.3%, *P* = 0.16). There was no significant difference in prevalence in any other metabolic risk factor in either gender barring a reduction in dyslipidemia in men over time (63.8% *vs* 52.9%, *P* < 0.01).

On age‐standardization, prevalence of obesity and elevated waist circumference significantly increased in women and decreased in men, T2DM prevalence increased in men but not women, and all other metabolic risk factors reduced in both genders (Table S3). Adequate diet and healthy lifestyle significantly reduced in both genders, consumption of takeaway food at least once per week increased in both genders, and participation in sufficient physical activity reduced in women but remained stable in men (Table S2).

### Changing risk factor profile and fibrosis risk in non‐alcoholic fatty liver disease

**Figure 4:**
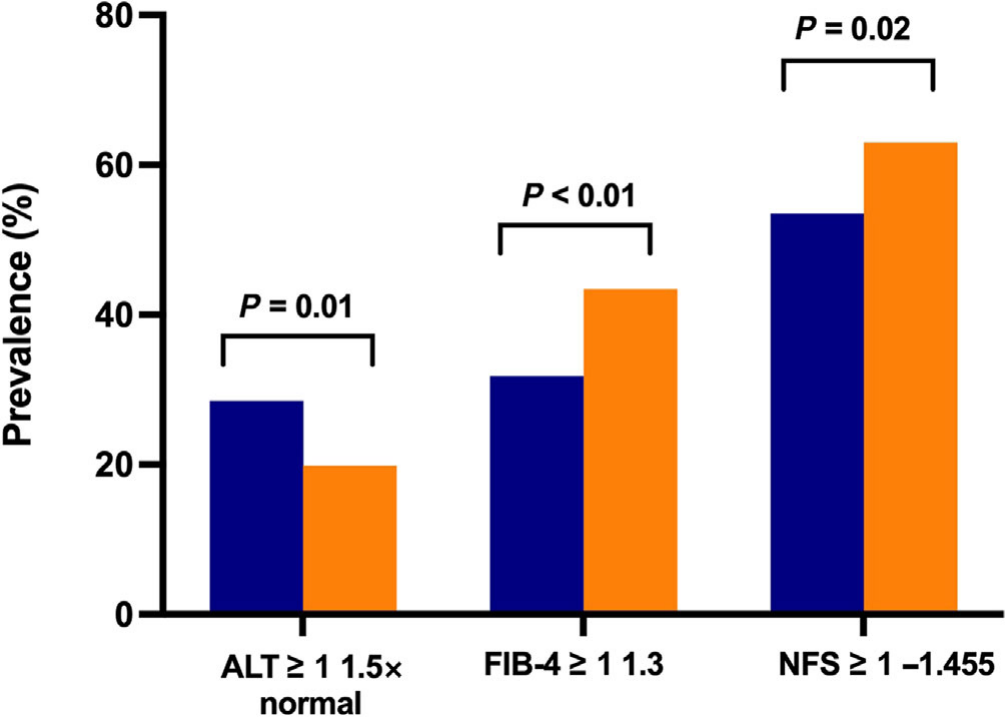
Changes in non‐invasive tests in those with NAFLD. 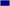, CrossRoads 1; 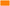, CrossRoads 2.

NAFLD subjects were older and more likely to live in a rural town and had completed secondary school or beyond, with no change in ethnic or racial background over the 15‐year study period (Table 3). There was a significant increase in mean BMI and waist circumference over time among participants with NAFLD. Dyslipidemia was the most common associated metabolic risk factor (≥ 70%), followed by hypertension (≥ 65%), metabolic syndrome (≥ 60%), while far fewer had T2DM (≈20%) (Table 3). Fewer NAFLD participants had an adequate diet and led a healthy lifestyle over time, with proportion consuming takeaway food one or more times per week numerically higher and physical activity stable between cohorts. Despite a reduction in proportion with elevated alanine aminotransferase (ALT) (28.5% *vs* 19.8%, *P* = 0.01), a greater number of patients had indeterminate or high‐risk for liver fibrosis on NITs in CR‐2 (FIB‐4: 43.4% *vs* 31.8%, *P* < 0.01; NFS: 63.0% *vs* 53.5%, *P* = 0.02) (Table 3, Fig. 4). When accounting for age‐specific cut‐offs,^17^ there was no longer a rise in indeterminate or high‐risk for fibrosis (Fig. S1).

**Table 3:** Difference in baseline demographics in NAFLD participants between CrossRoads studies

Variable	CrossRoads 1 (*n* = 340)	CrossRoads 2 (*n* = 273)	*P*‐value
***Demographic***			
Male	192 (56.5%)	136 (59.8%)	0.10
Age, years	54.2 (15.0)	61.2 (15.4)	**<0.01**
Australian‐born	303 (89.4%)	231 (84.6%)	0.08
White ethnic background	329 (97.1%)	256 (93.8%)	0.05
Rural location*	117 (34.4%)	148 (54.2%)	**<0.01**
Education secondary school and beyond	138 (40.9%)	164 (60.3%)	**<0.01**
***Clinical***			
Weight, kg	92.7 (14.5)	95.0 (17.3)	0.07
BMI, kg/m^2^	32.5 (5.3)	33.5 (5.9)	**0.03**
BMI, kg/m^2^			0.43
< 25	8 (2.4%)	4 (1.5%)	
25 to < 30	113 (33.2%)	81 (29.7%)	
≥ 30	219 (64.4%)	188 (68.9%)	
Waist circumference, cm	107.2 (10.3)	109.8 (12.1)	**<0.01**
Elevated waist circumference	293 (86.2%)	241 (88.3%)	0.44
Hypertension	236 (69.4%)	179 (65.8%)	0.34
Dyslipidemia	262 (80.1%)	186 (70.5%)	**<0.01**
Type 2 diabetes mellitus	46 (13.5%)	41 (15.5%)	0.49
Metabolic syndrome	213 (62.6%)	169 (62.8%)	0.96
***Lifestyle factors***			
Smoking status	49 (14.4%)	25 (9.4%)	0.15
Current smoker	138 (40.6%)	107 (40.4%)	
Ex‐smoker	153 (45.0%)	133 (50.2%)	
Non‐smoker			
Physical activity, minutes/week	278 (261)	240 (189)	0.10
Adequate physical activity	145 (68.7%)	115 (64.2%)	0.35
Adequate diet	79 (23.2%)	39 (14.7%)	**<0.01**
Takeaway food ≥ once per week	98 (28.8%)	96 (36.2%)	0.05
Healthy lifestyle	42 (19.9%)	22 (12.3%)	**0.04**
***Laboratory measured risk factors***			
GGT, U/L	33 (24, 51)	29 (20, 44)	**<0.01**
ALT, U/L	25 (17, 36)	26 (20, 35)	0.90
ALT ≥ 1.5× upper limit of normal**	97 (28.5%)	54 (19.8%)	**0.01**
AST, U/L	25 (21, 30)	23 (20, 29)	0.42
FIB‐4	1.24 (0.92)	1.29 (0.64)	0.45
FIB‐4 categorical			**<0.01**
< 1.30	232 (68.2%)	138 (56.6%)	
1.30 to 2.67	92 (27.1%)	98 (40.2%)	
> 2.67	16 (4.7%)	8 (3.3%)	
NAFLD Fibrosis Score (NFS)	−1.306 (1.485)	−1.041 (1.335)	**0.03**
NFS categorical			0.08
<−1.455	158 (46.5%)	87 (37.0%)	
−1.455 to 0.676	152 (44.7%)	124 (52.8%)	
>0.676	30 (8.8%)	24 (10.2%)	

All continuous parameters presented as mean (SD) except GGT, ALT, AST presented as median (IQR); all categorical parameters presented as *n* (%).

ALT, alanine aminotransferase; AST, aspartate aminotransferase; BMI, body mass index; FIB‐4, fibrosis‐4 index; GGT, gamma‐glutamyl transferase; NAFLD, non‐alcoholic fatty liver disease.

† Shepparton/Mooroopna considered regional, Benalla/Cobram/Seymour considered rural.

‡ Upper limit normal = 30 U/L men and 20 U/L women.

## Discussion

From these population‐based cross‐sectional studies with equivalent methodology conducted 15 years apart in regional Victoria, we found that the crude and age‐standardized/sex‐standardized prevalence of NAFLD has risen by 18.7% and 9.3%, respectively, with NAFLD now affecting nearly two in five adults. Moreover, we found this prevalence rise is associated with an increase in the prevalence of obesity and frequency of takeaway food consumption, reduction in consumption of adequate fruit and vegetables and participation in sufficient physical activity and occurred most steeply in women. Finally, up to 6 in 10 NAFLD patients have indeterminate or high‐risk for liver fibrosis on serum‐based NITs, necessitating additional assessment of fibrosis.

Despite the widely accepted high global disease burden of NAFLD, few research efforts have gone into ascertaining the prevalence over time, with most epidemiologic studies focused on point prevalence. Our study findings are consistent with existing literature from overseas, in particular the US, in showing an increase in NAFLD prevalence over the past two decades. Ruhl et al. utilized multiple cycles of the National Health and Nutrition Examination Survey (NHANES) in the US to demonstrate the prevalence increased by 68% from 18.2% in 1988–1991 to 30.6% in 2011–2012, with the greatest rise occurring in the first decade (57% relative increase), compared with an attenuated increase at the turn of the millennium (7% relative increase).^19^ Other studies have also shown prevalence of waist circumference^19^, ^20^ and BMI^20^ have increased in parallel with that of NAFLD, while hypertension has remained stable and lipid parameters decreased with time.^20^ The worrying prevalence increase has also been demonstrated in global meta‐analyses.^1^, ^2^

An important novel finding from our study was that the rise in prevalence of NAFLD was greater in women than men, even though men had higher NAFLD prevalence across timepoints. In contrast, the only other study reporting on the gender difference in prevalence change over time reported a greater increase in men than women in Japan.^21^ In our study, the predominant associated factor for difference again appears to be obesity and visceral adiposity, as prevalence of obesity and elevated waist circumference demonstrably rose in women by 38% and 35%, respectively, but not significantly in men. However, a difference in longitudinal prevalence trend of overweight and obesity between genders was not born out in the Global Burden of Diseases 2013 Obesity Collaboration study,^6^ and thus, further investigation is required to confirm our findings and clarify the factors leading to these differences. This is particularly relevant as a 2021 meta‐analysis by Balakrishnan et al. investigating the relationship between gender and prevalence of NAFLD and biopsy‐proven NASH and advanced fibrosis/cirrhosis reported the risk of advanced fibrosis or cirrhosis to be 37% higher in women than men^22^; this was not found in the current study (data not shown).

Standardized prevalence of participation in a healthy lifestyle, defined by sufficient physical activity and adequate consumption of fruit and vegetables, decreased over time, while frequency of takeaway food consumption increased. There are clearly defined links between physical inactivity and caloric excess with obesity and insulin resistance,^23^, ^24^ and over the past four decades the developed world has seen an adoption of a more sedentary lifestyle and consumption of more energy dense foods. Further, takeaway food has been associated with obesity, thought secondary to increased portion sizes and consumption of energy dense food.^23^ This is a possible contributor to the prevalence rise in NAFLD in this study. However, in a prior study conducted from CrossRoads, a direct relationship between obesity prevalence and takeaway consumption was not established but rather the increased obesity prevalence between cohorts was linked to physical inactivity, lack of employment, consumption of fat‐based spreads and advancing age.^25^ In any respect, the parallel rise in obesity and NAFLD with divergent uptake of a healthy lifestyle provide impetus for strong public health messaging about the adverse consequences of physical inactivity and caloric excess.

Finally, our finding that the prevalence of indeterminate or advanced fibrosis/cirrhosis on widely utilized NITs for staging NAFLD fibrosis—FIB‐4 and NFS—is high, requires particular attention. A commonly encountered approach to staging liver fibrosis in NAFLD is the sequential use of serum‐based NITs, followed by confirmatory tests such as transient elastography (i.e. FibroScan®) in the event of indeterminate or high results.^16^ Adopting this strategy, we found that 4 in 10 and 6 in 10 NAFLD patients met the indication for further elastographic assessment of fibrosis based on FIB‐4 and NFS, respectively, amounting to 3.10 million to 4.66 million adult Australians requiring referral for transient elastography or similar elastographic evaluation when using the more common cut‐offs irrespective of age. Given a significant portion of these patients will require referral to specialized hepatology care, often in tertiary centers, the economic burden of this cannot be understated. This is even more pertinent for the studied cohort in regional Victoria, where access to specialty care is limited compared with metropolitan centers.

For the first time, we have been able to evaluate the change in NAFLD prevalence in Australia with granular data determining the clinical and lifestyle factors implicated in this rise over time, and to ascertain high‐risk groups for targeted population‐based screening or intervention studies. However, our study also has limitations. For one, hepatosteatosis was determined by FLI rather than ultrasonography, limiting sensitivity for detection. However, this approach is endorsed by international societies for case‐ascertainment in population‐based studies.^11^ Furthermore, this impacted on statistical testing for determining more direct correlation between obesity and NAFLD. Second, we did not have data on soft drink consumption and coffee intake in both studies to assess their change with time. Third, our population was from regional Victoria, impacting the generalizability to metropolitan populations in Australia and internationally. Third, ethnicity‐specific cut‐offs were not adopted for definition of overweight, albeit this is unlikely to significantly impact on reported prevalence of overweight in this population given the small proportion of included participants who identified their ethnicity as Asian with only a small absolute increase in those of Asian descent between the two timepoints. Fourth, the finding that dyslipidemia was the most associated metabolic risk factor with NAFLD needs to be taken in the context that triglycerides forms part of FLI calculation. However, dyslipidemia was also the most prevalent risk factor among the entire cohort, so the association is likely to be true. Finally, two prevalence points do not constitute a trend, and further studies on repeated cross‐sectional population databases are warranted to assess for trend analysis.

In conclusion, we have demonstrated a concerning increase in NAFLD prevalence by around 10% over the past 15 years in a regional part of Australia, associated by a dramatic increase in the prevalence of obesity, particularly among women. Moreover, a significant proportion of NAFLD subjects have potentially at‐risk liver fibrosis. Taken together, these findings indicate NAFLD is a growing major public health problem that will significantly impact on health services that need to risk‐assess and manage the alarmingly high number of affected persons. As such, a concerted public health policy is required *a priori* to establish efficient preventative measures to both curb the current trend and manage the increasing clinical demand.

## Supporting information


**Table S1.** Differences in clinical features between included and excluded cases from analysis.
Table S2. NAFLD prevalence between rural and regional centres.
Table S3. Age‐standardized prevalence of metabolic risk factors and lifestyle covariates between men and women over time.

**Figure S1.** Changes in FIB‐4 and NAFLD Fibrosis Score using age‐specific cut‐offs.

## Data Availability

The data that support the findings of this study are available on request from the corresponding author.
