# Supplementary material for: Non‐alcoholic fatty liver disease prevalence in Australia has risen over 15 years in conjunction with increased prevalence of obesity and reduction in healthy lifestyle
Source: J Gastroenterol Hepatol. 2023 Aug 12;38(10):1823–31. doi: 10.1111/jgh.16314 (PMC10946623; doi:10.1111/jgh.16314)
Supplement: Supplementary file 1 — Table S1. Differences in clinical features between included and excluded cases from analysis. Table S2. NAFLD prevalence between rural and regional centres. Table S3. Age‐standardized prevalence of metabolic risk factors and lifestyle covariates between men and women over time. [file JGH-38-1823-s002.docx]

**Supplementary Appendix.**

**
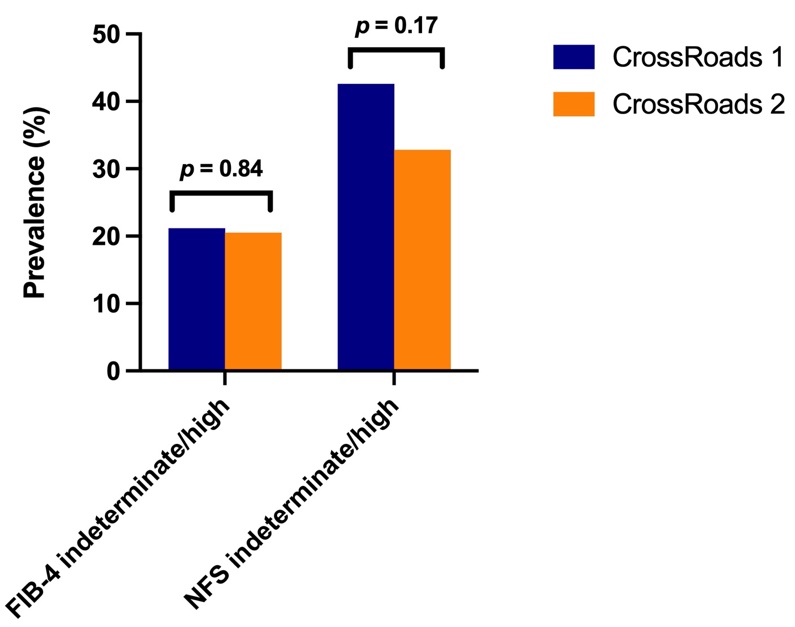
**

**Supplementary Figure 1.** Changes in FIB-4 and NAFLD Fibrosis Score using age-specific cut-offs

|  | **CrossRoads 1** | | |  | **CrossRoads 2** | | |
| --- | --- | --- | --- | --- | --- | --- | --- |
| **Variable** | **Included cases (n=1040)** | **Excluded cases (n=8)** | ***p*-value** |  | **Included cases (n=704)** | **Excluded cases (n=43)** | ***p-*value** |
| Male gender | 460 (44.2%) | 2 (25%) | 0.48 |  | 314 (44.6%) | 20 (46.5%) | 0.81 |
| Age, years | 52.5 (40.9, 65.8) | 40.8 (37.3, 44.4) | 0.30 |  | 62 (50.5, 70.5) | 58 (52, 70) | 0.29 |
| Born in Australia | 910 (87.9%) | 8 (100%) | 0.61 |  | 596 (84.8%) | 38 (95.0%) | 0.10 |
| White | 1011 (97.4%) | 8 (100%) | 1.0 |  | 654 (92.9%) | 19 (95.0%) | 1.0 |
| BMI, kg/m^2^ | 26.8 (24.1, 30.0) | 24.9 (24.9, 24.9) | 0.35 |  | 27.7 (24.8, 31.2) | 27.4 (24.5, 31.3) | 0.72 |
| Hypertension | 578 (55.6%) | 3 (37.5%) | 0.48 |  | 384 (54.9%) | 16 (42.1%) | 0.12 |
| Dyslipidaemia | 653 (63.8%) | 3 (37.5%) | 0.15 |  | 402 (57.8%) | 24 (61.5%) | 0.65 |
| Type 2 diabetes mellitus | 76 (7.3%) | 1 (12.5%) | 0.46 |  | 64 (9.4%) | 6 (15.8%) | 0.20 |
| Metabolic syndrome | 342 (32.9%) | 1 (14.3%) | 0.44 |  | 252 (36.3%) | 11 (30.6%) | 0.49 |
| Alcohol excess | 166 (16.0%) | 0 (0%) | 0.37 |  | 99 (14.1%) | 3 (11.1%) | 1.0 |
| Physical activity, minutes/week | 210 (120, 315) | 180 (180, 180) | 0.67 |  | 210 (120, 308) | 180 (60, 570) | 0.21 |
| Adequate diet | 228 (22.0%) | 3 (37.5%) | 0.39 |  | 133 (19.5%) | 4 (11.4%) | 0.29 |
| GGT, U/L | 22 (16, 33) | 12 (7, 17) | **<0.01** |  | 21 (15, 34) | 37 (25, 48) | 0.05 |
| ALT, U/L | 20 (14, 28) | 23 (13, 32) | 0.16 |  | 22 (17, 31) | 30 (20, 38) | 0.29 |
| Total cholesterol, mmol/L | 5.2 (4.5, 5.9) | 6.2 (4.4, 7.9) | 0.35 |  | 5.0 (4.2, 5.7) | 5.2 (4.9, 6.2) | 0.54 |
| LDL, mmol/L | 3.1 (2.5, 3.7) | 3.4 (2.1, 4.6) | 0.19 |  | 2.8 (2.2, 3.5) | 3.2 (2.2, 3.6) | 0.14 |
| HDL, mmol/L | 1.4 (1.2, 1.7) | 2.1 (1.9, 2.3) | **0.04** |  | 1.4 (1.2, 1.7) | 1.4 (1.1, 2.0) | 0.75 |
| Fasting glucose, mmol/L | 5.0 (4.7, 5.5) | 4.5 (4.5, 4.5) | 0.12 |  | 5.0 (4.7, 5.4) | 5.3 (4.8, 5.5) | 0.15 |
| FIB-4 | 1.13 (0.77, 1.52) | 0.78 (0.71, 0.84) | 0.22 |  | 1.19 (0.83, 1.63) | 1.23 (0.97, 1.52) | 0.68 |
| NAFLD Fibrosis Score | -1.730 (-2.642, -0.757) | -2.210 (-2.448, -1.971) | 0.49 |  | -1.477 (-2.370, -0.590) | -1.739 (-2.738, -0.848) | 0.24 |
| All continuous variables presented as median (IQR); all categorical variables presented as n(%)  BMI = body mass index; GGT = gamma-glutamyl transferase; ALT = alanine aminotransferase; LDL = low density lipoprotein; HDL = high density lipoprotein; FIB-4 = fibrosis-4 index | | | | | | | |

**Supplementary Table 1.** Differences in clinical features between included and excluded cases from analysis

|  | **Crude Prevalence** | | | **Standardised Prevalence** | | |
| --- | --- | --- | --- | --- | --- | --- |
|  | **Rural** | **Regional** | ***p*-value** | **Rural** | **Regional** | ***p*-value** |
| **CrossRoads 1** | 33.4 | 32.3 | 0.72 | 32.4 (27.4-37.3) | 31.1 (27.4-34.7) | **<0.01** |
| **CrossRoads 2** | 39.7 | 37.8 | 0.61 | 37.1 (30.9-43.4) | 34.4 (29.0-39.7) | **<0.01** |
| Data presented as percentage with 95% confidence intervals presented for standardised prevalence  Rural = Benalla, Cobram, Seymour; Regional = Shepparton-Mooroopna | | | | | | |

**Supplementary Table 2.** NAFLD prevalence between rural and regional centres

| **Variable** | **Men** | | | **Women** | | |
| --- | --- | --- | --- | --- | --- | --- |
|  | **CrossRoads 1** | **CrossRoads 2** | ***p*-value** | **CrossRoads 1** | **CrossRoads 2** | ***p*-value** |
| Obesity | 31.3 (27.5-35.0) | 30.3 (24.0-36.7) | **<0.01** | 28.6 (24.5-32.7) | 36.6 (31.6-41.7) | **<0.01** |
| Elevated waist circumference | 49.6 (45.5-53.8) | 44.6 (38.7-50.4) | **<0.01** | 51.0 (46.5-55.5) | 64.9 (59.7-70.0) | **<0.01** |
| Hypertension | 61.8 (58.1-65.5) | 58.4 (51.4-65.3) | **<0.01** | 49.5 (45.8-53.2) | 43.1 (38.3-47.8) | **<0.01** |
| Dyslipidaemia | 58.9 (54.7-63.2) | 49.0 (42.4-55.6) | **<0.01** | 64.4 (60.2-68.6) | 58.5 (53.2-63.9) | **<0.01** |
| Type 2 diabetes mellitus | 8.8 (6.3-11.2) | 10.8 (7.2-14.4) | **<0.01** | 6.4 (4.2-8.6) | 4.7 (2.8-6.6) | **<0.01** |
| Metabolic syndrome | 34.8 (30.7-38.9) | 33.9 (28.5-39.4) | **<0.01** | 32.2 (28.2-36.2) | 30.4 (25.7-35.2) | **<0.01** |
| Adequate physical activity | 66.3 (60.7-71.9) | 65.9 (58.1-73.7) | 0.15 | 64.5 (59.2-69.7) | 59.6 (52.7-66.6) | **<0.01** |
| Adequate diet | 15.0 (11.7-18.2) | 11.0 (6.6-15.4) | **<0.01** | 26.7 (23.0-30.5) | 21.9 (17.3-26.4) | **<0.01** |
| Takeaway consumption ≥ once per week | 23.8 (20.4-27.2) | 44.5 (37.6-51.5) | **<0.01** | 25.5 (21.7-29.3) | 33.7 (28.8-38.5) | **<0.01** |
| Healthy lifestyle | 13.6 (9.9-17.2) | 9.7 (5.1-14.3) | **<0.01** | 21.0 (17.0-25.0) | 18.1 (13.5-22.8) | **<0.01** |
| Data presented as percentage (95% confidence interval) | | | | | | |

**Supplementary Table 3.** Age-standardized prevalence of metabolic risk factors and lifestyle covariates between men and women over time
