# Supplementary figures and images for: Non‐alcoholic fatty liver disease prevalence in Australia has risen over 15 years in conjunction with increased prevalence of obesity and reduction in healthy lifestyle
Source: J Gastroenterol Hepatol. 2023 Aug 12;38(10):1823–31. doi: 10.1111/jgh.16314 (PMC10946623; doi:10.1111/jgh.16314)

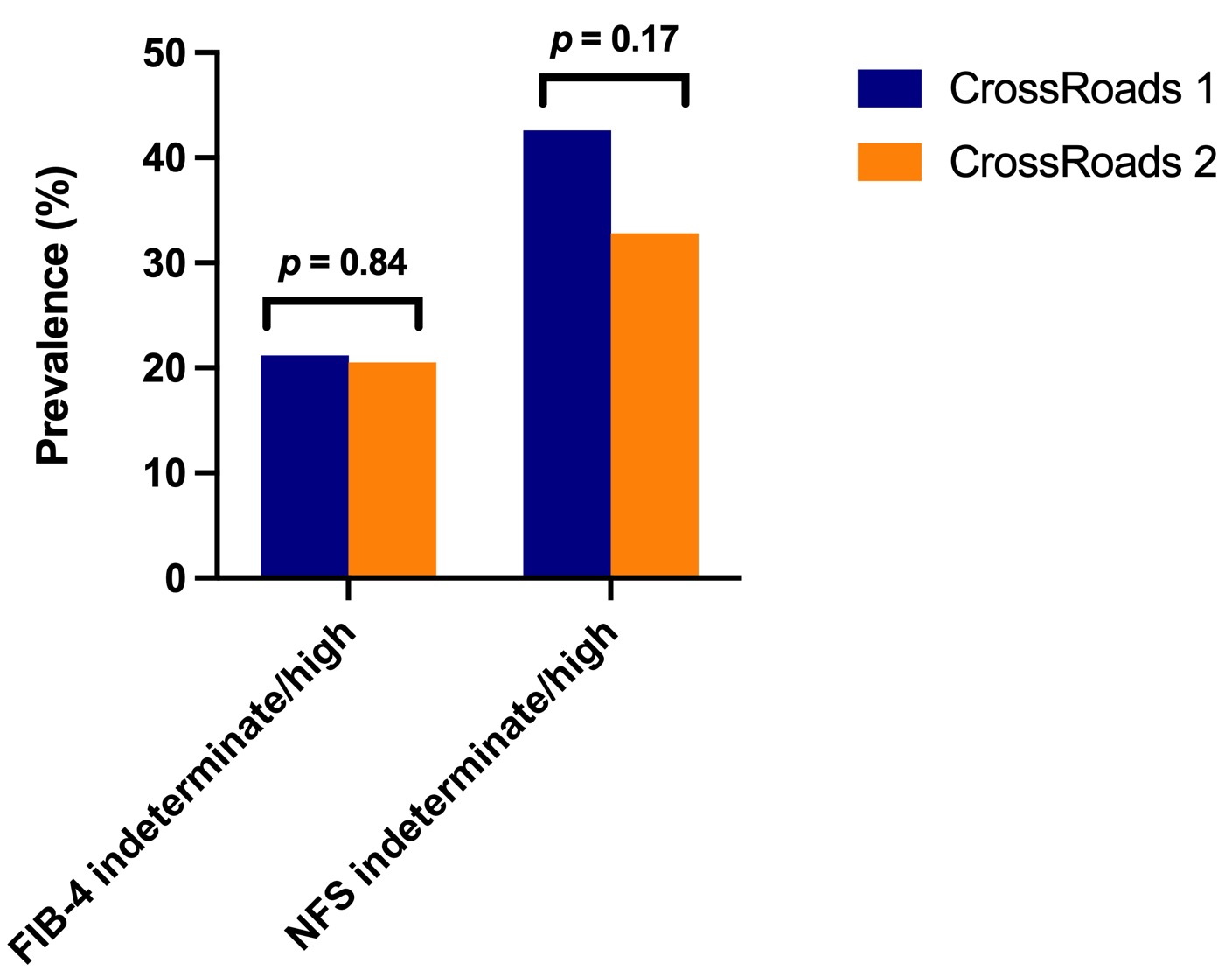

Supplement: Supplementary file 2 — Figure S1. Changes in FIB‐4 and NAFLD Fibrosis Score using age‐specific cut‐offs. [file JGH-38-1823-s001.jpg]
